# Goal Salience Affects Infants’ Goal-Directed Gaze Shifts

**DOI:** 10.3389/fpsyg.2012.00391

**Published:** 2012-10-09

**Authors:** Ivanina Henrichs, Claudia Elsner, Birgit Elsner, Gustaf Gredebäck

**Affiliations:** ^1^Department of Psychology, University of PotsdamPotsdam, Germany; ^2^Department of Psychology, Uppsala UniversityUppsala, Sweden

**Keywords:** anticipation, eye movement, salience, infant, action understanding

## Abstract

Around their first year of life, infants are able to anticipate the goal of others’ ongoing actions. For instance, 12-month-olds anticipate the goal of everyday feeding actions and manual actions such as reaching and grasping. However, little is known whether the salience of the goal influences infants’ online assessment of others’ actions. The aim of the current eye-tracking study was to elucidate infants’ ability to anticipate reaching actions depending on the visual salience of the goal object. In Experiment 1, 12-month-old infants’ goal-directed gaze shifts were recorded as they observed a hand reaching for and grasping either a large (high-salience condition) or a small (low-salience condition) goal object. Infants exhibited predictive gaze shifts significantly earlier when the observed hand reached for the large goal object compared to when it reached for the small goal object. In addition, findings revealed rapid learning over the course of trials in the high-salience condition and no learning in the low-salience condition. Experiment 2 demonstrated that the results could not be simply attributed to the different grip aperture of the hand used when reaching for small and large objects. Together, our data indicate that by the end of their first year of life, infants rely on information about the goal salience to make inferences about the action goal.

## Introduction

The ability to anticipate other people’s actions is crucial for the planning and control of one’s own actions in accordance with the actions of others. Already at the age of 6–9 months, infants are able to predict others’ goal-directed actions (Southgate et al., 2010; Kanakogi and Itakura, 2011). Moreover, around their first year of life, they anticipate a variety of different manual actions such as reaching (Cannon and Woodward, 2012), placing objects inside a container (Falck-Ytter et al., 2006), or everyday feeding actions (Gredebäck and Melinder, 2010).

A considerable amount of literature indicates a close relationship between infants’ ability to anticipate observed actions and their motor ability of the same actions (Gredebäck and Kochukhova, 2010; Gredebäck and Melinder, 2010). To illustrate, Kanakogi and Itakura (2011) demonstrated that 6-month-olds’ emerging motor ability to perform grasping actions corresponded to their ability to anticipate the goal of observed grasping actions. Similarly, Falck-Ytter et al. (2006) found that 12- but not 6-month-olds were able to anticipate the goal of a manual action, consisting of transporting balls to a container. Because 12- but not 6-month-olds have extensive experience with reaching and placing actions, the authors interpreted these data as evidence for the link between motor experience and action understanding. Additionally, Gredebäck and Melinder (2010) found that 12- but not 6-month-olds’ anticipatory performance of observed feeding actions was correlated with their lifetime experience being fed.

Apart from motor experience, there are other influence factors as well. For instance, when observing other people act on objects, infants are commonly faced with scenes where multiple objects with different shape and size are available (Ambrosini et al., 2011). Indeed, information about the properties of the goal is crucial for the planning and control of one’s own actions (Castiello, 2005). Research on human prehension indicates that object parameters such as size, shape, and weight have a great impact on the execution of grasping actions in adults (Smeets and Brenner, 1999; Castiello, 2005). Recently, it was found that the properties of the goal have also an impact on adults’ goal anticipations (Eshuis et al., 2009). To illustrate, in Ambrosini et al.’s (2011) study, adults observed action events in which a hand was reaching for and grasping one of two differently sized goal objects. In one condition, the hand was pre-shaped so that adults could use the grip information in order to predict the goal of the ongoing action (a whole hand grip for the big object and a precision grip for the small object). In the no-shape condition, the hand moved with a closed fist configuration to the goal objects. Results showed that in the pre-shape condition, adults looked at the correct goal object ahead of time, with earlier gaze-arrival times at the large goal object compared to the small goal object. Interestingly, even in the no-shape condition, adults looked ahead of time toward the large object. This effect was ascribed to the salience of the large object. Analogously, Eshuis et al. (2009) presented adults with videos in which a human agent was moving a toy frog toward a bucket. In one condition the transporting action was followed by end-effects: when the toy entered the bucket water ripples were shown and frog croaking was played. In a control condition, there were no end-effects. Eshuis et al. (2009) found an earlier gaze-arrival time at the action goal in the end-effects condition compared to the no-effects condition, indicating an impact of goal salience on adults’ goal anticipations.

To date, little is known about the degree to which the properties of the goal influence infants’ online assessment of others’ actions. For instance, Cannon et al. (2012) used similar set-up to that applied in Falck-Ytter et al.’s (2006) study, presenting 12-month-old infants with events in which a human agent was placing three balls into a bucket. Cannon et al. (2012) found later gaze-arrival times compared to those found for 12-month-olds in Falck-Ytter et al.’s (2006) study. More specifically, in Falck-Ytter et al.’s study, infants’ gaze shifts passed the threshold of 0 ms, indicating that they were able to look at the goal ahead of time, whereas infants’ gaze shifts in Cannon et al.’s study did not. Cannon et al. attributed this effect to a procedural difference between the two studies. Namely, while in the first study there were end-effects accompanying the arrival of the ball into the bucket (an artificial sound was played and a face pattern imposed on the bucket), there were no such end-effects in the latter study.

Although these studies indirectly support the notion that goal salience might have an impact on goal anticipations, this idea has not yet been directly addressed in infants. Hence, the following experiments seek to investigate the impact of goal salience on infants’ ability to anticipate reaching actions. In two experiments, we demonstrate that the visual salience of the goal object has an impact on infants’ goal anticipations. This effect cannot be simply attributed to the different grip aperture of the hand when reaching for small and large objects. Our results indicate that action prediction in infancy might be influenced by the properties of the goal such as the size of the goal object.

## Experiment 1

In Experiment 1, we investigated the influence of the visual salience of the goal on 12-month-old infants’ ability to anticipate reaching actions performed by a human agent. We presented videos in which a human hand reached for one goal object. In the high-salience condition, the hand reached for a large goal object, whereas in the low-salience condition, it reached for a small goal object. In order to investigate infants’ goal anticipations, we measured their predictive gaze shifts (Gredebäck et al., 2010). If infants use information about the salience of the action goal, then they should show earlier gaze-arrival times in the high-salience condition compared to the low-salience condition (Ambrosini et al., 2011). If goal salience does not have an impact on infants’ processing of reaching actions, then gaze performance between conditions should not differ. To our knowledge, this is the first infant study to directly investigate the influence of goal salience on infants’ goal anticipation.

### Material and methods

#### Participants

The final sample consisted of 24 12-month-old infants, 12 in each condition (6 females in each condition). The mean age was 365 days (*SD* = 7) in the high-salience condition and 366 days (*SD* = 7) in the low-salience condition. An additional two infants were excluded because of fussiness or calibration failure. Parents were contacted by phone and signed a consent form prior to their participation. The study was approved by the Regional Ethic Committee according to the 1964 Declaration of Helsinki. Each family was given a gift certificate (approximately 10 Euro) for participation.

#### Stimuli and apparatus

Gaze was measured with a Tobii T120 near infrared eyetracker (Tobii, Stockholm, Sweden) with an infant add-on; monitor size 17″; accuracy 0.5°, sampling rate 60 Hz. A standard five-point calibration was used (Gredebäck et al., 2010). Infants were presented with videos (25.5 × 19.1 visual degrees) of a human hand reaching for one goal object placed on a table.

**Figure 1:**
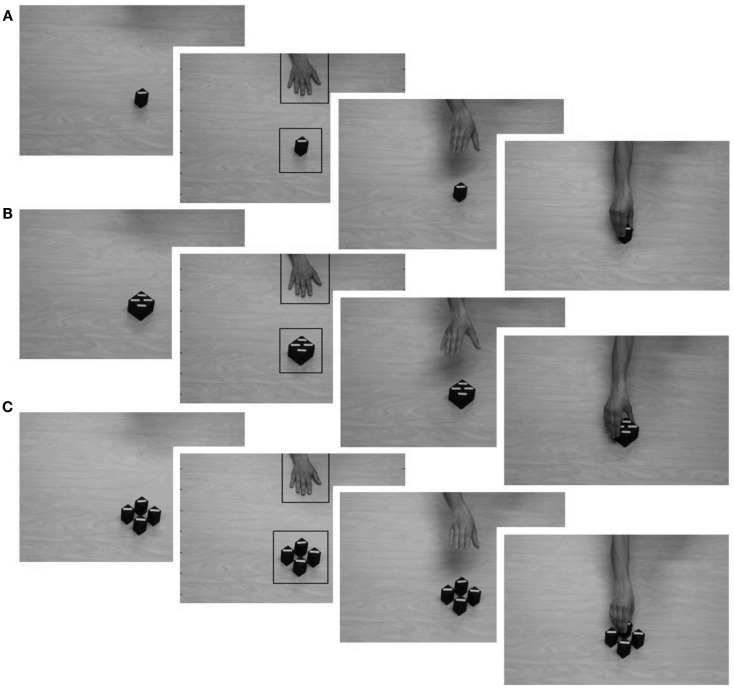
**Snapshots of the action sequence in each video, depicting the beginning of each movie, the hand resting on the table, and the reaching and grasping action in the low-salience (A) and high-salience (B) condition in Experiment 1 and the low-salience (A) and high-salience (C) condition in Experiment 2**. Areas of interest (AOIs) for the hand and for each goal object are marked with black rectangles.

Each video began with a still frame giving a view of a wooden table top, filmed from above, with either one small or one large rectangular blue object positioned in the middle of the screen. After 500 ms, a human hand entered the scene from above and moved to the upper middle of the table (500–960 ms). It rested motionless on the table (960–1800 ms) and then reached for the goal object (1800–2960 ms). The hand grasped the object (2960–3760 ms), and rested on the object for the last 1240 ms (see Figure 1). Each video lasted for approximately 5000 ms. There were two videos, one in which the hand reached for the small goal object (see Figure 1A) and one in which the hand reached for the large goal object (see Figure 1B).

#### Procedure

During the experiment, infants sat on their caregivers’ lap in a curtained experimental room and performed a calibration procedure first. Then infants were presented with videos of either the high-salience or low-salience condition, interleaved with brief animations designed to reorient their attention to the screen. There were 14 trials in each condition. Each family spent approximately 15 min in the lab.

#### Data reduction and analysis

Infants’ predictive gaze shifts during the reaching action were analyzed. Two areas of interest (AOIs) were created to cover the hand (5.8 visual degrees horizontal extension) and the goal object (5.6 visual degrees horizontal extension) the hand was reaching for (see Figure 1). The size of the AOI covering the goal object was identical in both conditions. Infants first had to fixate the hand for at least 200 ms and then shift their gaze to the goal AOI. Mean gaze-arrival times were calculated by subtracting the time when infants first looked inside the goal AOI from the time when the observed hand first entered the same AOI. Thus, positive numbers refer to a gaze-arrival before the hand arrived at the goal AOI, value of zero indicates gaze-arrival at the same time as the hand and negative numbers a gaze-arrival after the hand arrived at the goal AOI. Gaze shifts were classified as functionally predictive if they occurred before the hand entered the goal AOI, that is, if the 95% confidence interval with lower boundary for each group mean was above 0. This threshold is conservative and ensures that infants actually look at the correct location ahead of time (Gredebäck et al., 2010). It has previously been used in the majority of action prediction studies in infancy (Falck-Ytter et al., 2006; Gredebäck and Melinder, 2010; Kanakogi and Itakura, 2011).

Data from each action were included if infants attended to the hand for at least 200 ms and fixated the goal AOI no later than 1000 ms after the hand had entered the goal AOI. All included infants had minimum five out of 14 valid trials. Mean gaze-arrival times were aggregated over trials 1–9. The last five trials were excluded because of lack of attention. Mean gaze-arrival times were compared between conditions using independent *t* tests. Effect sizes were calculated using Cohen’s *d*. Both linear and curvilinear regression analyses were run to analyze learning effects across trials 1–9 in each condition. The regression line with the highest explained variance (linear or curvilinear) was reported and displayed in Figure 3.

### Results

#### Overall gaze-arrival time

**Figure 2:**
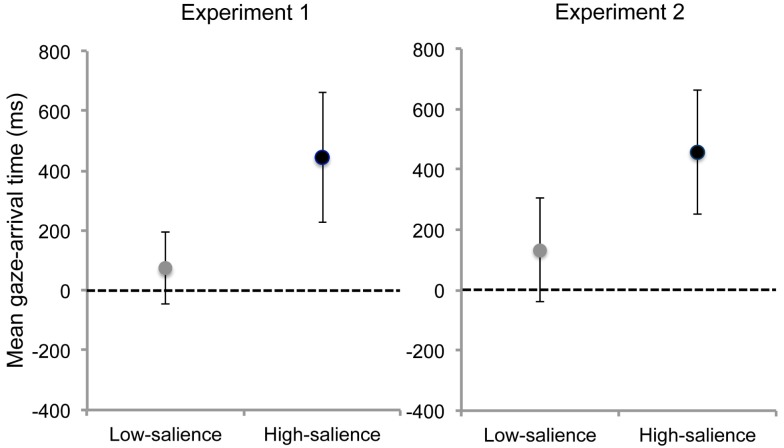
**Mean gaze-arrival times (in ms) relative to the hand’s arrival time for the aggregated means of trials 1–9 for the high- and low-salience condition in Experiments 1 and 2**. Error bars depict 95% confidence intervals. The horizontal line represents the threshold of 0 ms and differentiates predictive from reactive gaze shifts. Values above 0 correspond to earlier arrival of gaze relative to the arrival of the hand at the goal area.

A comparison of the aggregated mean gaze-arrival times of trials 1–9 revealed a significant difference between conditions, *t*(15.62) = 2.52, *p* = 0.023, *d* = 1.27. Infants in the high-salience condition showed significantly earlier mean gaze-arrival times than infants in the low-salience condition (see Figure 2).

Infants’ mean gaze-arrival times in the high-salience condition passed the threshold of 0 ms, 95% CI [182, 704], whereas infants’ mean gaze-arrival times in the low-salience condition did not, 95% CI [−49, 192], indicating that only infants in the prior group were able to fixate the goal object ahead of time.

#### Learning effects

**Figure 3:**
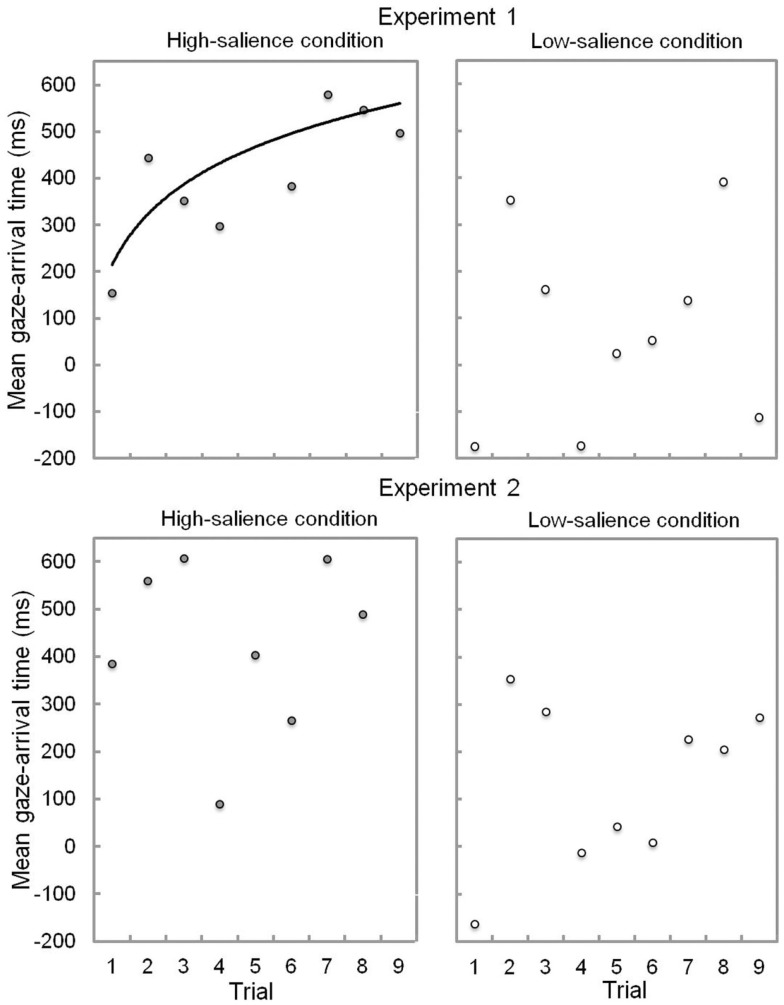
**Mean gaze-arrival times (in ms) over trials 1–9 in the high- and low-salience condition in Experiments 1 and 2**. The solid curve depicts the regression line with most explained variance. Note: no significant regression line could be fitted for the low-salience condition in Experiment 1 as well as for the high- and low-salience conditions in Experiment 2.

Within the first two trials, infants in the high-salience condition learned to predict the goal object of the reaching action and their performance improved throughout the experimental session. This learning effect is best described using the logarithmic function, *y* = 197.59ln(*x*) + 181.39, expressing a rapid improvement of gaze performance over the course of trials (see Figure 3), $R_{adj}^{2}$= 0.52, *F*(1, 8) = 9.84, *p* = 0.02. By contrast, infants in the low-salience condition did not show improvement of performance throughout the experimental session (see Figure 3).

### Discussion

The purpose of Experiment 1 was to investigate the impact of the visual salience of the goal object on infants’ goal anticipations of observed reaching actions. We found that 12-month-old infants exhibited gaze shifts significantly earlier when the observed hand reached for the large goal object as compared to when it reached for the small goal object. Additionally, only infants in the high-salience condition were able to look at the goal ahead of time, fixating the goal object before the hand arrived at the goal AOI. Therefore, our data indicate that by the end of their first year of life, infants rely on information about the properties of goal objects to make inferences about the action goal. To our knowledge, this is the first infant study to directly demonstrate this effect during the observation of reaching actions.

In Falck-Ytter et al.’s (2006) study, 12-month-olds looked at the goal ahead of time when observing a human agent transporting balls into a bucket, indicating predictive gaze shifts. By contrast, same-aged infants in Cannon et al.’s (2012) study were not able to fixate the goal object ahead of time when observing comparable transporting actions. Cannon et al. attributed these differences to the goal being more salient in Falck-Ytter et al.’s study. In our study, mean gaze-arrival times in the high-salience condition (443 ms) were comparable to those in previous infant studies on action prediction, indicating that the salience of the goal in this condition was similar to the goal salience in these studies (Falck-Ytter et al., 2006; Gredebäck and Melinder, 2010; Kanakogi and Itakura, 2011). By contrast, when infants observed actions during which the hand reached for a small goal object, they were not able to fixate the goal object ahead of time, resulting in later mean gaze-arrival times (72 ms), comparable to that found by Cannon et al.

In the current investigation, infants in the high-salience condition rapidly learned to track the reaching action in a predictive manner within only a few trials, whereas infants in the low-salience condition did not so. Interestingly, the learning curve in the high-salience condition was highly similar to that found by Kochukhova and Gredebäck (2007) for occluded non-social action events. It seems that the salience of the goal affects infants’ learning to anticipate goal-directed actions throughout the experimental session.

Taken together, our data indicate that the properties of the goal object have an impact on infants’ goal anticipations. Particularly, the visual salience of the goal object contributed to the overall predictive gaze shifts in the high-salience condition. This notion is supported by Ambrosini et al.’s (2011) study in which adults exhibited earlier gaze-arrival times when the hand was reaching for a large goal object as compared to when it was reaching for a small goal object. However, Ambrosini et al. attributed this effect to the pre-shaping of the hand rather than to the visual salience of the goal object. More specifically, the hand was pre-shaped to a whole hand grip to reach for the large object and to a precision grip to reach for the small object, the latter requiring more time to be processed than the prior (Ambrosini et al., 2011). The idea that observers use information about the hand shape when processing others actions, is also supported by other studies. For instance, Fischer et al. (2008) demonstrated that adults rapidly inferred the goal object of an observed grasping action from the shape of the actor’s hand in a reaction-time study. Moreover, already at the age of 6 months, infants are able to infer the size of the goal object from the pre-shaping of the grasping hand (Daum et al., 2009). Although we used a power grip in both conditions, the configuration of the hand actually differed between conditions. More specifically, the aperture between all fingers and the thumb was larger in the high-salience condition than in the low-salience condition. Thus, it might be that the earlier gaze-arrival times in the high-salience condition were not only due to the size of the goal object but also to the wider grip of the hand. This possibility was addressed in Experiment 2.

## Experiment 2

In Experiment 2, we tested the assumption that the grip aperture accounts for the difference in gaze performance between conditions in Experiment 1. We presented videos in which a human hand reached for one small goal object in both conditions. In the high-salience condition, there were four small objects available, which preserved the overall higher visual salience of the goal area in this condition. The low-salience condition was identical to that of Experiment 1. Thus, in Experiment 2, all infants saw the hand reaching for a small goal object resulting in a narrow grip in both conditions. If the pre-shaping of the hand had the greatest impact on infants’ predictive gaze shifts in Experiment 1, we would expect no difference in the mean gaze-arrival times between conditions in Experiment 2. If however, the visual salience of the goal was crucial for the predictive gaze shifts, then infants in the high-salience condition should still show earlier gaze-arrival times than infants in the low-salience condition.

### Material and methods

#### Participants

The final sample consisted of 24 12-month-old infants, 12 in each condition (6 females in each condition). None of the infants had participated in Experiment 1. The mean age was 365 days (*SD* = 7) in the high-salience condition and 364 days (*SD* = 9) in the low-salience condition. An additional three infants were excluded because of fussiness or calibration failure.

#### Stimuli and apparatus

The stimuli and apparatus were identical to that of Experiment 1 with the following exception. In the high-salience condition, infants saw a movie in which the hand was grasping for one of four small rectangular objects positioned next to each other, forming a rectangular form. The total size of the four small objects was comparable to that of the large goal object used in Experiment 1. The hand reached for and grasped the nearest of the four small objects which was exactly on the same position as the small object in the low-salience condition (see Figure 1C). In the low-salience condition, infants were presented with an action event identical to that in Experiment 1 (see Figure 1A). Thus in both movies, the hand was shaped to a narrow power grip when reaching for the goal object.

#### Procedure, data reduction, and analysis

The procedure, data reduction, and analyses were identical to that of Experiment 1.

### Results

#### Overall gaze-arrival time

There was a significant difference between conditions, *t*(22) = 2.40, *p* = 0.025, *d* = 1.02. Infants in the high-salience condition showed significantly earlier mean gaze-arrival times than infants in the low-salience condition (see Figure 2). Mean gaze-arrival times did not differ between Experiment 1 and 2 neither for the high-salience condition, *t*(22) = 0.08, *p* = 0.93, *d* = 0.03, nor for the low-salience condition, *t*(22) = 0.52, *p* = 0.61, *d* = 0.22.

Infants’ gaze-arrival times in the high-salience condition passed the criterion of 0 ms, 95% CI [252, 664], whereas infants’ gaze-arrival times in the low-salience condition did not, 95% CI [−43, 301], suggesting that only infants in the first group were able to fixate the goal object prior to the arrival of the hand at the goal AOI.

#### Learning effects

There was no linear or curvilinear regression line fitting the data, indicating no learning effects in Experiment 2 (see Figure 3).

### Discussion

In Experiment 2, we addressed the possibility that the higher gaze performance in the high-salience condition compared to the low-salience condition in Experiment 1 was due to the wider grip of the hand rather than to the visual salience of the large object. Although the grip aperture was identical in both conditions, we found a significant difference between conditions, indicating that infants in the high-salience condition (four small objects available) exhibited gaze shifts much earlier than infants in the low-salience condition (one small object available). Moreover, the mean gaze-arrival times in the high-salience condition in Experiment 2 (*M* = 458 ms) were similar to those in Experiment 1 (*M* = 443 ms). Furthermore, just like in Experiment 1, only infants in the high-salience condition were able to look at the goal object ahead of time, before the hand arrived at the goal AOI. As soon as the goal is highly salient, infants anticipate the goal of a reaching hand in a functional way no matter if the hand is pre-shaped in a wide or narrow power grip. Thus, it seems that gaze performance is not only affected by subtle motor information (see Falck-Ytter, 2012), but also by the object-related properties such as the size of the goal object. To our knowledge, this is the first study to disentangle the contribution of these two factors on infants’ goal-directed gaze shifts.

Interestingly, in Experiment 2, we failed to find any learning effects during the experimental session. Because most infant studies on action prediction either did not find or they did not report learning effects (Falck-Ytter et al., 2006; Gredebäck et al., 2009; Kanakogi and Itakura, 2011; Cannon et al., 2012), it is difficult to explain the presence of learning effects in Experiment 1 and the absence of those in Experiment 2. It might be that a larger sample size is required in order to find clearly visible learning effects. Alternatively, although the overall size of the goal area in the high-salience condition was kept similar between experiments, in Experiment 1, the hand was approaching the goal object pre-shaped in a wide power grip, whereas in Experiment 2 the reaching hand was pre-shaped in a narrow power grip. It might be that the learning effect in the high-salience condition in Experiment 1 was influenced by both the salience of the goal object and the grip aperture used during the reach. Future research should address the factors influencing infants’ learning when observing others’ manual actions.

## General Discussion

This study is the first to demonstrate that infants’ goal-directed gaze shifts are modulated by the visual salience of the goal object. Twelve-month-olds in Experiment 1 exhibited predictive gaze shifts significantly earlier when the observed hand reached for a large as compared to a small goal object, which is consistent with Ambrosini et al.’s (2011) findings with adults. Interestingly, Ambrosini et al. attributed the difference in gaze performance in their study to the pre-shaping of the hand rather than to the visual salience of the goal object. Although we kept the grip aperture constant between conditions in Experiment 2, infants in the high-salience (large goal area) condition still fixated the goal earlier than infants in the low-salience (small goal area) condition. Hence, our data indicate that it is the visual salience of the goal object what accounted for differences in gaze performance between conditions.

One difference between the two studies was that in the present investigation the reaching hand was always shaped to a power grip, only slightly varying its aperture depending on the size of the goal object. By contrast, in Ambrosini et al.’s (2011) study the hand was pre-shaped to a power or precision grip depending on the to-be-grasped object. The authors argued that the precision grip needs more time to be processed compared to the power grip. It might be that infants’ processing of a power grip is independent from the exact distance between the fingers and the thumb. Additionally, in the adult study, the large and the small objects were both present during the reaching action. Thus, another likely explanation is that information about the exact kinematics of the handgrip is crucial in situations, where multiple objects are present and the goal of the reaching action cannot be predicted in advance (Falck-Ytter, 2012). This idea is supported by an adult study in which participants were able to predict the goal of an ongoing action from the kinematics of the moving hand without prior knowledge of the agent’s intention (Rotman et al., 2006). As soon as a single goal object is available, infants might only pay attention to global kinematic information from the moving arm, neglecting more subtle motor information such as the grip aperture.

It might be that infants’ earlier gaze-arrival times in the high-salience condition were driven by a general selective process modulated by the size of the goal object. This notion is supported by adult studies indicating that large objects capture attention in visual search tasks (Proulx, 2010). Thus, it is possible that a larger object captures more attention leading to earlier gaze-arrival times irrespective of the action type observed. Indeed, in Ambrosini et al.’s (2011) study, adults exhibited predictive gaze shifts to the large object even when the hand moved to the goal objects with a closed fist configuration. This effect was ascribed to the higher visual salience of the large object. Analogously, in Eshuis et al.’s (2009) study, apart from the human agent condition, there was a self-propelled condition, in which the frogs moved to the bucket on their own. Just as in the human agent condition, the transporting action was either followed by end-effects or not. Eshuis et al. found that as soon as end-effects accompany the transporting action, adults exhibit earlier gaze shifts irrespective of the action type, indicating a great impact of goal salience on adults’ goal anticipations.

Alternatively, recent research indicates that the mirror neuron system (MNS) is involved in the processing of others’ goal-directed actions (Rizzolatti and Craighero, 2004; Gallese et al., 2009). In their seminal study, Flanagan and Johansson (2003) demonstrated that when observing others’ manual actions, adults exhibit similar predictive eye movements to those found when they execute the action themselves. This phenomenon is described by a direct matching mechanism within the MNS, in which observed actions are mapped onto the observer’s motor representation of the same action (Rizzolatti et al., 2001; Rizzolatti and Craighero, 2004). Evidence for the direct matching hypothesis was also found in infants (Rosander and von Hofsten, 2011). Moreover, a considerable amount of research indicates that infants’ ability to anticipate observed actions is tightly linked to their own motor experience with the same actions (Falck-Ytter et al., 2006; Gredebäck and Kochukhova, 2010; Gredebäck and Melinder, 2010; Kanakogi and Itakura, 2011). For instance, in Falck-Ytter et al.’s (2006) study, 6- and 12-month-old infants observed action sequences, consisting of a transporting balls to a container. While in one condition the balls were transported by a human agent, in another condition they moved on their own. Results indicated that 12- but not 6-month olds were able to fixate the goal ahead of time, but only when the human agent performed the action. Because 12- but not 6-month-olds have extensive experience with transporting actions, Falck-Ytter et al. interpreted these data as evidence for the link between motor experience and action understanding. Furthermore, Kanakogi and Itakura (2011) found that 6- but not 4-month-old infants were able to anticipate grasping actions and that infants’ gaze performance corresponded to their emerging motor ability to perform grasping actions. Additionally, in control conditions including non-functional and non-human actions, they tracked those actions in a reactive manner. Similarly, Kochukhova and Gredebäck (2010) demonstrated that 6-month-olds anticipate that food is brought to the mouth, while combing actions and self-propelled spoons were tracked in a reactive manner. Together these studies demonstrate that infants’ ability to predict others’ actions is modulated by their motor experience with the same actions.

However, all of the above-mentioned studies only varied the type of the action, keeping the goal salience constant between conditions. By contrast, in the present investigation, we presented the same reaching action in both groups, varying the size of the goal object between conditions. As a result, 12-month-old infants were only able to anticipate the goal of the reaching action when the goal was highly salient. By comparison, infants in the low-salience condition failed to track the reaching action in a predictive manner. This is a surprising result given the fact that by the end of their first year of life, infants have gained extensive experience with reaching actions and are therefore supposed to be able to anticipate the reaching actions of others (Rosander and von Hofsten, 2011; Cannon et al., 2012). Thus, our data extend previous findings, suggesting that infants’ action prediction is not only modulated by motor experience but also by the properties of the goal. This is in line with what was found by Falck-Ytter et al. (2006) and Cannon et al. (2012) who used similar action sequences in their studies. Namely, in the presence of end-effects, 12-month-olds in Falck-Ytter et al.’s study were able to predict the goal of the transporting action in a functional way, whereas in the absence of such effects in Cannon et al’s study, infants failed to functionally predict the action goal. Hence, infants might not only need motor experience with a particular action, but also salient goals and end-effects in order to reliably predict those actions.

To sum up, this is the first infant study to find a direct evidence for the impact of goal salience on infants’ goal anticipations of observed reaching actions. More specifically, our data suggest that in a simple reaching action setting, a highly salient goal facilitates infants’ gaze shifts from the reaching hand to the goal object, enabling them to look at the goal object ahead of time. By contrast, in the case of low-salience, infants fail to track the reaching action in a predictive manner. It might be that a highly salient goal draws infants’ attention irrespective of the action type observed, indicating a general selective process. However, given the evidence from previous research, it is more likely that goal salience interacts with infants’ motor experience with the observed action. Future research should disentangle the role of these factors, varying both the action type and the salience of the goal. Only when we take into consideration the complex structure of predictive gaze shifts, we can understand how infants learn about the actions of others.

## Conflict of Interest Statement

The authors declare that the research was conducted in the absence of any commercial or financial relationships that could be construed as a potential conflict of interest.
